# Relationship between nurses’ critical thinking disposition and patient safety incident reporting: The mediating role of patient safety culture in a comprehensive nursing service ward

**DOI:** 10.1371/journal.pone.0315679

**Published:** 2024-12-12

**Authors:** Nam-Yi Kim, Sung-Jung Kwak

**Affiliations:** 1 Department of Nursing, Konyang University, Daejeon, Republic of Korea; 2 Department of Nursing, Howon University, Gunsan, Republic of Korea; Alexandria University Faculty of Nursing, EGYPT

## Abstract

At present, patient safety nursing by nurses is important and the risk of patient safety incidents is high. However, the comprehensive nursing service ward in Korea has no guardians. To prevent patient safety incidents and its recurrence, it is necessary to accurately report patient safety incidents. Patient safety incident reporting may be different depending on an individual’s critical thinking disposition and patient safety culture (organization, department, individual). This study was a descriptive survey and aimed to suggest ways to improve the reporting of patient safety incidents in Korea. The study participants were 130 nurses working in the comprehensive nursing service ward of a Korean university hospital. From October 5–18, 2023, we conducted a survey of nurses’ critical thinking dispositions, patient safety culture, and patient safety incident reporting. The mediating effect of patient safety culture on the relationship between critical thinking disposition and patient safety incident reporting was analyzed using PROCESS Macro Model 4. The results show that the patient safety incident report of nurses in the comprehensive nursing service ward was related to nurses’ critical thinking disposition and the nursing department’s patient safety culture. In particular, it was found that the department’s patient safety culture had a mediating effect (β = 0.11, 95% CI = 0.01~0.22) on the relationship between critical thinking disposition and patient safety incident reports. To encourage patient safety incident reports in nurses in the comprehensive nursing service ward, it is necessary to improve the organizational culture of the department (presenting free opinions or problems) and to develop strategies to promote critical thinking among nurses.

## Introduction

In Korea, the number of hospitalized patients has increased rapidly due to the growing older adult population, which has increased the burden of family caregiving and the economic cost of hiring caregivers. To solve this problem, comprehensive nursing services are being implemented, where professional nursing staff provide total nursing care to patients without the need for guardians or caregivers [1]. In addition to traditional nursing duties, nurses in the comprehensive care service ward are expected to perform non-specialized tasks such as fecal management, assisting with meals, and supervising nursing assistants [2]. They also have a critical responsibility to detect and prevent patient safety issues around the clock in systems where caregivers and guardians are not present [3]. However, due to the recent increase in hospitalization of patients with dementia, older adult patients, and critically ill patients, the risk of safety incidents such as falls and pressure ulcers is increasing, and nurses in all-inclusive care wards face the greatest challenges in patient safety liability [4].

To prevent such patient safety incidents and prevent their recurrence, patient safety incidents must be reported accurately. Therefore, a patient safety reporting system has been established to systematically collect data through voluntary reporting and analysis [5]. Enabling patient safety incident reporting requires critical thinking to quickly identify patient safety incidents, explore root causes, and act quickly to address and resolve them [6]. Critical thinking disposition is the ability to make careful and accurate assessments or judgments about a patient’s condition [7]; it is an essential skill for nurses in comprehensive care units, where skillful, multidimensional nursing care is required. Nurses with high levels of critical thinking have been shown to perform better clinically [8]. These skills can be utilized in situations where there is a risk of patient safety issues arising, allowing them to serve as patient safety stewards.

Patient safety can be achieved primarily by creating a high-level culture of patient safety, where everyone in the healthcare organization considers patient safety to be of utmost importance. Patient safety culture is the product of individual and organizational perceptions, attitudes, values, competencies, and behavioral patterns that determine an organization’s commitment to and proficiency in safety management [9]. A positive and carefully designed organizational patient safety culture can improve patient safety by encouraging nurses to report any incidents and errors [10]. Previous studies have shown that higher perceptions of patient safety culture are associated with higher levels of patient safety event reporting [9, 11]; hence, patient safety culture can be predicted as a variable that influences patient safety event reporting. In particular, previous studies have shown that the subdomains of patient safety culture, i.e., organizations, departments, and individuals, have different effects on patient safety nursing activities [2]. Therefore, understanding how the subdomains of patient safety culture play a role in patient safety incident reporting can aid in developing intervention strategies to activate patient safety incident reporting.

As discussed above, nurses’ reporting of patient safety incidents in comprehensive nursing service units may be positively influenced by nurses’ critical thinking disposition and patient safety culture.

However, the relationship between critical thinking dispositions, patient safety culture, and patient safety incident reporting has not been studied to date. Furthermore, it is important to consider that nurses’ critical thinking disposition is a factor that influences patient safety culture; thus, we can predict the mediating effect of patient safety culture on the relationship between nurses’ critical thinking disposition and patient safety incident reporting in comprehensive nursing service units.

Therefore, this study aims to examine the mediating effect of patient safety culture on the relationship between critical thinking disposition, patient safety culture, and patient safety incident reporting among nurses in a comprehensive nursing service unit. Further, this study seeks to determine the role of the subdomain factors of patient safety culture. The findings of this study can be used as a basis for a program to increase patient safety incident reporting by nurses in comprehensive care units.

## Material and methods

### Research design

This study uses a descriptive survey to determine the mediating effect of patient safety culture on the influence of critical thinking disposition on patient safety incident reporting among nurses in a comprehensive care unit.

### Participants and data collection

Study participants were nurses working in comprehensive nursing service wards of a university hospital in Korea using convenience sampling method. The inclusion criteria were: 1) nurses working in the nursing and care integration service ward, 2) those with more than six months of work experience, and 3) those who understood the purpose of the study and agreed to participate voluntarily. The exclusion criteria were: 1) not working in a nursing-care integrated service ward, 2) working experience of less than six months, and 3) not agreeing to participate in the study. The number of participants was determined using G*Power 3.1.9 based on an effect size of 0.15, a significance level of 0.05, and a power of 0.8 in an F-test using G*Power 3.1.9. The minimum required sample size was 127, and the number of participants was 141, considering a 10% dropout rate. The researcher personally visited and explained the purpose of the study to the director of nursing, obtained confirmation of institutional research consent for data collection, and obtained approval to proceed with the study. The researcher explained the necessity and purpose of the study to the subjects and distributed the questionnaire only if they agreed in writing to participate in the study.

A total of 141 respondents completed the survey, and 130 data were used in the final analysis, excluding 11 respondents who had more than 10% of the survey content missing (7.9% dropout rate).

### Instruments

#### Critical thinking disposition

Critical thinking disposition was measured using the Critical Thinking Disposition Measurement Tool developed by Insoo Kwon et al [12]. The instrument consists of 35 questions and is organized into eight sub-dimensions: intellectual integration, challenge, creativity, openness, objectivity, prudence, truth-seeking, and inquiry. Each question is answered on a 5-point Likert scale, ranging from 1 (not at all) to 5 (very much so). Higher scores indicate a higher propensity for critical thinking. The reliability of the instrument was Cronbach’s α = .89 when it was developed and Cronbach’s α = .90 in this study.

#### Patient safety culture

Patient safety culture was measured using the Korean patient safety culture perception measurement tool of Lee Soon-Kyo [13]. The tool consists of three dimensions: organizational, departmental, and individual, with a total of 35 questions. The subfactors of the organizational dimension are leadership, patient safety policies and procedures, and patient safety improvement system. The subfactors of the departmental dimension are teamwork and non-punitive environment. The subfactors of the individual dimension are patient safety knowledge and attitudes and patient safety priorities. Each item is scored on a 5-point Likert scale ranging from 1 (not at all true) to 5 (very true), with higher scores indicating a higher perception of patient safety culture. The reliability of the instrument was Cronbach’s α = .93 in Lee’s study [13] and Cronbach’s α = .94 in this study.

#### Patient safety incident reporting

Patient safety incident reporting was measured using the Patient Safety Incident Reporting Tool developed by Kim et al. [14]. The tool is a 13-item instrument with three subfactors: concerns about utilizing evaluation, beliefs about improvement effectiveness, and intention to report. Each question is answered on a 5-point Likert scale ranging from 1 (not at all) to 5 (very much so). Higher scores indicate more positive attitudes toward reporting patient safety events. The reliability of the instrument was Cronbach’s α = .80 in a study by Kim et al [14], and the reliability of this study was Cronbach’s α = .89.

#### Demographic characteristics of study participants

The demographic characteristics of the participants included sex, age, highest level of education, work experience, work department, work type, patient safety training within the past year, patient safety incident experience within the past year, and number of patient safety incident reports within the past year.

### Data analyses

The data collected in this study were analyzed using SPSS 25.0 (SPSS; IBM, Armonk, NY, USA) as follows. The participants’ general characteristics and the measured variables were analyzed using descriptive statistics. Differences in the degree of measured variables according to the participants’ general characteristics were analyzed by independent t-test, one-way ANOVA, Mann-Whitney U test, and Kruskal-Wallis test for non-parametric cases. Correlations between each measure were analyzed using Pearson’s correlation coefficients. The mediating effect of patient safety culture on the relationship between critical thinking disposition and patient safety incident reporting was analyzed using PROCESS Macro Model 4. In step 1, we analyzed whether critical thinking disposition has a significant effect on the mediating variable patient safety culture (organizational, departmental, and individual). In step 2, we analyzed whether critical thinking disposition and the mediating variable patient safety culture (organizational, departmental, and individual) influenced patient safety incident reporting. Step 3 analyzed the significance of the indirect effect of patient safety culture on the relationship between critical thinking disposition and patient safety incident reporting. The significance of the indirect effects was tested by estimating 95% confidence intervals using bootstrapping (10,000 resamples).

### Ethical considerations

This study was approved by the Institutional Review Board of K University, Daejeon, Korea (KYU 2023-06-018-001). In an effort to Obtaining the code of ethics and complying, the researcher explained the purpose and content of the study, confidentiality, voluntary participation in the study, and no disadvantages for withdrawing consent. If they voluntarily agreed to participate, they were asked to fill out the consent form, and then the questionnaire was distributed. The collected data is anonymized except for the researcher to ensure confidentiality, stored safely in the researcher’s personal space with a lock, and kept for three years for verification of the research data before being immediately shredded and disposed.

## Results

### Demographic characteristics of study participants

The participants comprised 124 (95.4%) females and 6 (4.6%) males. The most common age group was 25–29 years old (59 people, 45.4%). Additionally, 36 people (27.7%) were under 25 years old, 23 (17.7%) were 30–34 years old, and 12 (9.2%) were 35 years or older. Majority had a bachelor’s degree with 106 (81.5%), and the most participants had a work experience of “1–4 years” with 75 (57.7%), followed by “5–9 years” with 25 (19.2%), “10+ years” with 22 (16.9%), and “less than 1 year” with 8 (6.2%). The surgical ward accounted for 81 (62.3%) of the participants, and the third shift accounted for 126 (96.9%) of the participants (Table 1). In terms of patient safety incident-related characteristics, 125 (96.2%) of the participants reported having received patient safety education within one year, and 98 (75.4%) of the participants reported having experienced a patient safety incident within one year. The number of patient safety incidents reported within the past year was most common with 92 (70.8%) reporting 1–4 incidents, followed by 35 (26.9%) reporting none, and 3 (2.3%) reporting 5 or more incidents (Table 1).

**Table 1:** Demographic and sociological characteristics of participants (N = 130).

Characteristics	Categories	n	%	Mean±SD
**Sex**	Female	124	95.4	
	Male	6	4.6	
**Age (yr)**	< 25	36	27.7	27.94±4.71
	25–29	59	45.4	
	30–34	23	17.7	
	≥ 35	12	9.2	
**Educational degree**	Associate	17	13.1	
	Bachelor	106	81.5	
	Graduate	7	5.4	
**Total clinical** **career (yr)**	< 1	8	6.2	5.33±4.9
	1–4	75	57.7	
	5–9	25	19.2	
	≥ 10	22	16.9	
**Current work unit**	Surgical unit	81	62.3	
	Medical unit	49	37.7	
**Work shift**	Yes	126	96.9	
	No	4	3.1	
**Patient safety education experience (within 1 year)**	Yes	125	96.2	
	No	5	3.8	
**Patient safety incident experience (within 1 year)**	Yes	98	75.4	
	No	32	24.6	
**Patient safety incident report (number/within 1 year)**	None	35	26.9	1.28±1.37
	1–4	92	70.8	
	≥ 5	3	2.3	

### Differences in patient safety incident reporting by demographic characteristics

The differences in the means of patient safety event reporting according to the demographic characteristics of the participants were not statistically significant for sex (Z = -0.72, p = 0.474), age (F = 1.49, p = 0.220), education (χ2 = 2.18, p = 0.336), years of experience (χ2 = 5.81, p = 0.121), and work type (t = 0.33, p = 0.743). Differences in means were also not statistically significant for patient safety event education (Z = -1.48, p = 0.137), patient safety event experience (t = 0.89, p = 0.375), and number of patient safety event reports (χ2 = 0.20, p = 0.906) (Table 2).

**Table 2:** Differences in patient safety incident reporting according to demographic characteristics (*N* = 130).

Characteristics	Categories	Mean±SD	t or F Z or χ^2^	*p*
**Sex** *	Female	3.67±0.53	-0.72	0.474
	Male	3.95±0.82		
**Age (yr)**	< 25	3.66±0.55	1.49	0.220
	25–29	3.62±0.54		
	30–34	3.70±0.52		
	≥ 35	3.98±0.56		
**Educational degree** ^†^	Associate	3.55±0.58	2.18	0.336
	Bachelor	3.69±0.55		
	Graduate	3.86±0.45		
**Total clinical** **career (yr)** ^†^	< 1	3.62±0.49	5.81	0.121
	1–4	3.66±0.55		
	5–9	3.54±0.56		
	≥ 10	3.92±0.50		
**Current work unit**	Surgical unit	3.69±0.58	0.33	0.743
	Medical unit	3.66±0.49		
Work shift*	Yes	3.66±0.54	-1.48	0.137
	No	4.10±0.53		
Patient safety education experience (within 1 year)*	Yes	3.68±0.55	-0.24	0.808
	No	3.74±0.48		
**Patient safety incident experience (within 1 year)**	Yes	3.70±0.57	0.89	0.375
	No	3.60±0.45		
**Patient safety incident report (number/within 1 year)** ^†^	None	3.62±0.50	0.20	0.906
	1–4	3.70±0.56		
	≥ 5	3.77±0.61		

Notes:

* = Mann-Whitney U test;

^†^ = Kruskal-Wallis test.

### Correlations between metrics

The correlations between participants’ critical thinking dispositions, patient safety culture, and patient safety incident reporting variables were as follows (Table 3). Critical thinking disposition was positively and statistically significantly correlated with organizational patient safety culture professional respect (r = .54, p<0.001), departmental patient safety culture (r = .44 p<0.001), personal patient safety professionalization (r = .54, p<0.001), and patient safety incident reporting (r = .42, p<0.001). Organizational patient safety culture was positively and statistically significant with departmental patient safety culture (r = .64, p<0.001), individual patient safety culture (r = .65, p<0.001), and reporting of patient safety events (r = .39, p<0.001). Departmental patient safety culture was positively and statistically significant with individual patient safety culture (r = .77, p<0.001) and patient safety incident reporting (r = .47, p<0.001). Individual patient safety culture was positively correlated with patient safety incident reporting (r = .47, p<0.001) and was statistically significant.

**Table 3:** Correlation between critical thinking propensity, patient safety culture, and patient safety incident reporting (*N* = 130).

Variables	1	2	3	4	5
	r (*p*)	r (*p*)	r (*p*)	r (*p*)	r (*p*)
**1. Critical thinking propensity**	1				
**2. Organization patient safety culture**	.54 (<0.001)	1			
**3. Department patient safety culture**	.44 (<0.001)	.64 (<0.001)	1		
**4. Individual patient safety culture**	.54 (<0.001)	.65 (<0.001)	.77 (<0.001)	1	
**5. Patient safety incident report**	.42 (<0.001)	.39 (<0.001)	.47 (<0.001)	.47 (<0.001)	1

### Mediating effect of patient safety culture on the relationship between critical thinking disposition and reporting of patient safety incidents

To determine the mediating effect of patient safety culture on the effect of critical thinking disposition of nurses in comprehensive nursing service wards on patient safety incident reporting, simple regression analysis was used to check for multicollinearity and to analyze the direct and mediating effects between the variables (Table 4). As a result of testing the assumptions of the regression analysis, the tolerance of the variables ranged from 0.44 to 0.65, which is greater than 0.1, and the variance inflation factor ranged from 1.53 to 2.25, which is less than 10, indicating that there was no problem of multicollinearity. The Durbin-Watson index, an indicator of the independence of the error terms, was 1.89, which is within the standard range (1.8–2.2), confirming that there is no autocorrelation. The residual analysis showed that the normal P-P plot of the regression standardized residuals for patient safety incident reports was linear, the scatter plot showed that the distribution of the residuals was evenly distributed around zero, confirming the normality and equidistribution of the residuals, and the normality of the independent variables (skewness = 0.07–0.67, kurtosis = 0.29–1.55) was satisfied, confirming that the regression analysis was valid.

**Table 4:** Path coefficients and multiple parallel mediating effects of measurement variables.

**Direct effect**		**B**	**SE**	**β**	**t**	***p***	**95% CI**		**F(*p*)**	**R** ^**2**^
							**LLCI**	**ULCI**		
**Step 1**	X → M1	1.89	0.29	0.54	6.31	<0.001	1.30	2.48	52.80	0.29
	X → M2	0.51	0.32	0.44	6.87	<0.001	1.59	2.87	30.16	0.19
	X → M3	0.72	0.10	0.55	7.51	<0.001	0.53	0.91	56.39	0.31
**Step 2**	X → Y	0.31	0.14	0.21	2.24	0.027	0.04	0.58	12.27	0.28
	M1 → Y	0.03	0.14	0.03	0.23	0.818	-0.24	0.30		
	M2 → Y	0.31	0.15	0.25	2.05	0.043	0.01	0.62		
	M3 → Y	0.16	0.15	0.14	1.06	0.292	-0.13	0.45		
**Indirect effect**			**B**		**β**		**Boot SE**		**95% CI**	
									**Boot LLCI**	**Boot ULCI**
**Step 3**	X → M1 → Y		0.02		0.01		0.06		-0.11	0.12
	X → M2 → Y		0.16		0.11		0.06		0.01	0.22
	X → M3 → Y		0.11		0.08		0.12		-0.07	0.27
**Total effect**	**B**	**SE**	**β**	**t**	***p***		**95% CI**		**F(*p*)**	**R** ^**2**^
							**LLCI**	**ULCI**		
	0.60	0.12	0.41	5.15	<0.001	0.37	0.83	26.50	0.17	

**Notes:** X = critical thinking propensity; M1 = organization patient safety culture; M2 = department patient safety culture; M3 = individual patient safety culture; Y = patient safety incident report; CI = confidence interval; LLCI = low limit confidence interval; ULCI = upper limit confidence interval.

None of the demographic characteristics of the participants showed significant differences in patient safety incident reporting, so no control variables were used. The mediating effect of patient safety culture on the effect of critical thinking disposition on patient safety incident reporting by nurses in the comprehensive nursing service wards is shown in Table 4. In step 1, we tested whether the independent variable, critical thinking disposition, had a significant effect on the mediating variable, patient safety culture (organizational, departmental, and individual). Critical thinking disposition had a direct effect on organizational patient safety culture (β = 0.54, p<0.001), departmental patient safety culture (β = 0.44, p<0.001), and individual patient safety culture (β = 0.55, p<0.001), and was statistically significant. In step 2, we tested whether the independent variable, critical thinking disposition, and the mediating variable, patient safety culture (organizational, departmental, and individual), influenced the dependent variable, patient safety incident reporting. Critical thinking disposition had a direct and statistically significant effect on patient safety incident reporting (β = 0.21, p = 0.027) and departmental patient safety culture (β = 0.25, p = 0.043), but not on organizational patient safety culture (β = 0.03, p = 0.818) or individual patient safety culture (β = 0.14, p = 0.292). However, the indirect effects of organizational patient safety culture (β = 0.01, 95% CI = -0.11–0.12) and individual patient safety culture (β = 0.08, 95% CI = -0.07–0.27) on the relationship between critical thinking disposition and patient safety incident reporting were not statistically significant (Table 4).

**Fig 1:**
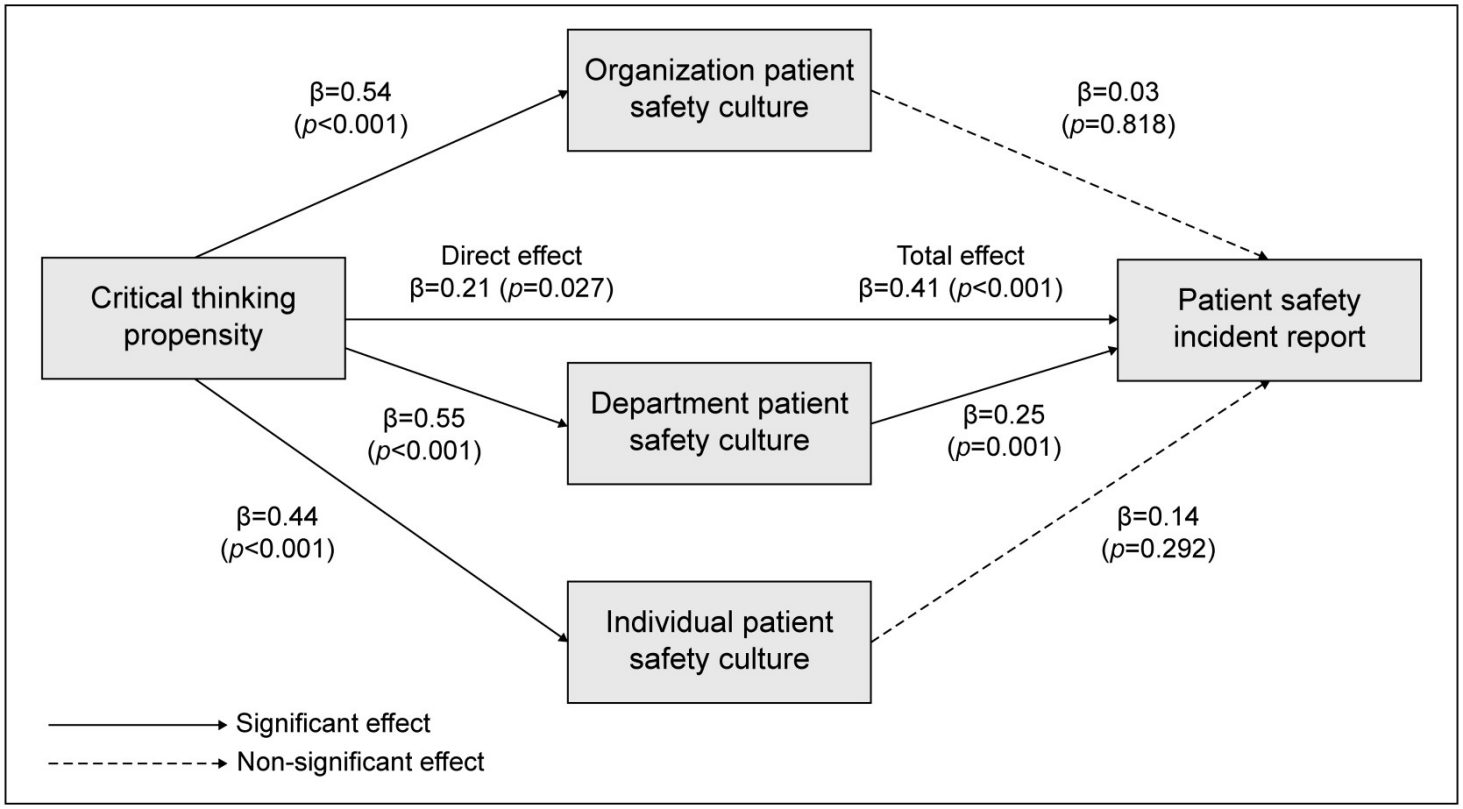
Mediating effect of patient safety culture on the relationship between critical thinking trends and patient safety accident reporting.

In other words, the critical thinking disposition of nurses in the comprehensive nursing service ward mediated the patient safety culture of the department to influence the reporting of patient safety incidents (Fig 1).

## Discussion

This study aimed to determine the relationship between critical thinking disposition, patient safety culture, and patient safety incident reporting among nurses in a comprehensive nursing service ward. Further, it seeks to determine the mediating effect of patient safety culture on the relationship between critical thinking disposition and patient safety incident reporting.

In this study, patient safety incident reporting was related to the general characteristics of the participants (sex, age, education, work experience, work department, and work type), patient safety education, patient safety incident experience, and the number of patient safety incident reports. The differences in the means were not statistically significant. The study participants were nurses in a comprehensive nursing service ward, and although there is no comparable study with the same participants, previous studies on nurses in Korea have shown that higher age and work experience are associated with higher levels of patient safety incident reporting [10, 15]. The increased work burden of providing total nursing care without a caregiver or guardian in comprehensive nursing service wards may have led to different results from previous studies of general nurses; thus, it is recommended that future studies of nurses in comprehensive nursing service wards must be conducted to promote the reporting of patient safety incidents.

There were positive correlations between critical thinking disposition, patient safety culture, and patient safety incident reporting. First, a higher critical thinking disposition among nurses in the comprehensive nursing service wards was associated with higher patient safety incident reporting. Although the studies on the relationship between nurses’ critical thinking disposition and patient safety incident reporting are sparse and cannot be compared, previous studies have shown that nurses’ critical thinking disposition is a significant predictor of patient safety competence [16] and is positively related to patient safety awareness [6] and attitudes [17]. This confirms the similarity of the results of this study, as patient safety competence and patient safety awareness and attitudes are predictors of patient safety incident reporting. Second, patient safety culture was also positively correlated with patient safety incident reporting, indicating that the higher the perception of patient safety culture among nurses in the comprehensive nursing service ward, the higher the rate of patient safety incident reporting. A previous study [10] also confirmed that nurses’ awareness of patient safety culture affects the reporting of patient safety incidents. Farokhzadian et al. [18], who explored the relationship between nurses’ perceptions of patient safety culture and patient safety event reporting in a foreign country, supported this finding by reporting that higher nurses’ perceptions of patient safety culture were associated with higher rates of patient safety event reporting. Finally, nurses’ critical thinking disposition was positively related to patient safety culture and had a direct effect on all three subscales of patient safety culture perception: organizational, departmental patient, and individual patient safety culture. Previous studies [19] have also shown that nurses’ critical thinking disposition positively influenced their perception of patient safety culture. Therefore, the positive relationship between nurses’ critical thinking disposition and their perception of patient safety culture should be considered to increase the reporting rate of patient safety incidents among nurses in nursing-care integrated service wards.

This study found that nurses’ critical thinking in the comprehensive nursing service ward influenced their perception of patient safety culture. The nurses’ critical thinking disposition directly influenced all subscales of their perception of patient safety culture, and the department’s patient safety culture mediated the relationship between critical thinking disposition and patient safety incident reporting. Dolatabadi and Ziaeirad [20] suggested that enhancing nurses’ critical thinking may help to promote patient safety culture, which may support the findings of this study on the relationship between critical thinking disposition and patient safety culture. Critical thinking disposition is the attitude of questioning generally accepted or socially recognized facts and acknowledging and questioning the possibility of error in oneself and others [21]. The critical thinking disposition of these nurses leads them to perform nursing activities for patient safety such as pressure ulcer management, medication administration, and infection control based on evidence rather than relying on existing knowledge, their own nursing experience, or the way they have always done things. In addition, by questioning their own and others’ potential for error, nurses will be able to prevent harm and errors by creating a culture of patient safety within the department. Therefore, it is necessary to form an organizational culture in which nurses can discuss with each other whenever they have doubts about commonly accepted or recognized facts in their work. Next, in this study, the patient safety culture of nurses in comprehensive nursing service wards was a variable that influenced the reporting of patient safety incidents, and previous studies have shown that the higher the perception of departmental patient safety culture and ward patient safety culture, the higher the intention to report patient safety culture [22]. In addition, by strengthening patient safety specialization, the reporting of patient safety incidents can be increased, emphasizing the need to strengthen patient safety culture [10]. This study found that departmental patient safety culture had a direct effect on patient safety incident reporting and a mediating effect on the relationship between critical thinking disposition and patient safety incident reporting; however, organizational patient safety culture and individual patient safety culture had no direct effect on patient safety incident reporting and no mediating effect. A departmental patient safety culture and the reinforcement of shared beliefs, values, and behavioral patterns about patient safety in the comprehensive nursing care unit to which the nurse belongs can increase the rate of reporting patient safety incidents, but neither organizational nor individual nurse patient safety culture led to higher rates of reporting patient safety events.

Previous studies have shown that factors related to individual patient safety culture include reduced workload, improved knowledge, and standardization of nursing processes [23], and organizational patient safety culture is related to hospital size and monthly working hours [24]. The number of nurses in Korea is 4.4 per 1000 population, which is about 2 times less than the OECD average of 8.0, and the resulting working hours are more than 60 hours per week, which may be due to excessive nursing workload and working hours.

Meanwhile, this is consistent with research showing that a positive and well-designed departmental patient safety culture encourages nurses to report errors [9], and a large study of 220,000 nurses in the United States found that departmental teamwork influences patient safety incident reporting [25]. Safety incidents in healthcare are most often caused by a lack of awareness of the safety culture and are more often the result of departmental issues than individual problems [26]. Therefore, rather than blaming the parties involved when a patient safety incident occurs, efforts should be made at the level of the medical institution to raise the level of patient Safety Culture in the department and increase the reporting behavior of patient safety incidents.

The results of this study suggest that the critical thinking disposition of nurses in an integrated care unit influences patient safety incident reporting through the culture of patient safety in the unit. Based on this, we believe that it is necessary to create an organizational culture in which nurses in comprehensive nursing service wards can strengthen their critical thinking disposition, which includes fairly evaluating the opinions of others in communication with other healthcare providers, exploring to make decisions and solve problems according to clinical situations, performing evidence-based nursing care, and maintaining systematic thinking about the work environment and situation, by establishing programs and creating an organizational culture where they can be actively expressed. In addition, I think it is necessary to establish a patient safety culture promotion program to check the degree of nurses’ awareness of patient safety culture and improve the deficiencies so that the subdomains of patient safety culture can function organically. This strategy of enhancing nurses’ patient safety expertise through enhanced critical thinking disposition will ultimately lead to nurses’ reporting of patient safety incidents.

An important significance of the results of this study is that it confirmed the effect of critical thinking disposition as an antecedent of patient safety culture. The significance of this study is that it suggests the need for future replication and expansion of the study because there are not many studies that have explored the relationship between critical thinking disposition and patient safety culture among nurses in comprehensive nursing service wards. In addition, hospitals and nursing managers may consider developing and applying critical thinking training programs for nurses to increase their awareness of patient safety culture. In addition, we believe that the results of this study suggest that nurses’ critical thinking disposition and attention to patient safety culture should be addressed as a strategy to promote patient safety incident reporting.

### Limitations

Since this study was conducted only on nurses in comprehensive nursing service wards in Korea, follow up studies in various medical settings are needed to increase the generalizability of the findings. As the various antecedent factors suggested in the patient safety culture model could not be considered together, it will be necessary to confirm them through future research. In particular, the patient safety incident reporting system may be different for each hospital, but this was not confirmed in this study; hence, it is necessary to determine the differences in the degree of patient safety incident reporting according to the reporting system in future research. In addition, we recommend a longitudinal study to identify the causal relationship of patient safety incident reporting and a study to develop interventions to strengthen critical thinking and patient safety culture.

## Conclusions

This study examined the effects of nurses’ critical thinking disposition and patient safety culture on patient safety incident reporting and the mediating effect of patient safety culture. The significance of this study is that it confirmed the effect of nurses’ critical thinking disposition as a variable that affects patient safety incident reporting and suggested that measures should be taken to promote patient safety culture through nurses’ critical thinking to activate patient safety incident reporting, In other words, to activate patient safety incident reporting by nurses in comprehensive nursing service wards, it is necessary to improve the organizational level to enable nurses to think critically, and it is necessary to develop strategies to induce nurses to think critically in the work process. Future research should include personal characteristics as antecedents of patient safety culture along with nurses’ critical thinking disposition, and it is suggested that the influence of each subdomain of patient safety culture on patient safety incident reporting should be repeated in various nursing organizations.
